# Supplementary material for: Complexome profiling of the Chlamydomonas psb28 mutant reveals TEF5 as an early PSII assembly factor
Source: Plant Cell. 2025 Mar 17;37(6):koaf055. doi: 10.1093/plcell/koaf055 (PMC12164590; doi:10.1093/plcell/koaf055)
Supplement: koaf055_Supplementary_Data [file koaf055_supplementary_data.zip › psb28_interactive.zip!/plots/intensity/qualityPlots/peptides/SCO1_Cre17.g732850.t1.2.html]

### Cre17.g732850.t1.2

Trivial name: SCO1  
SeqCoverage [median]: 32.60  
Mass: 34.33 kDa  
  
 

| Sequence | MissedCleavage | Mass [Da] | wtIntensity | muIntensity | Score |
| --- | --- | --- | --- | --- | --- |
| AASDVAPVSMR | 0 | 1102.54 | 0.00 | 992.81 | 82.29 |
| AMVAAGAAAAEPK | 0 | 1156.59 | 3638.42 | 4717.07 | 148.32 |
| LQQVTAK | 0 | 786.46 | 1258.38 | 1925.04 | 108.92 |
| LYTDQKLQQVTAK | 1 | 1534.84 | 462.42 | 0.00 | 36.30 |
| MIGLTGSLDK | 0 | 1033.55 | 0.00 | 2315.38 | 98.03 |
| NADAPTIAK | 0 | 899.47 | 552.44 | 5110.80 | 113.74 |
| SQQVVGQASVGGPFELTDQDGKPFSNK | 1 | 2819.37 | 0.00 | 1190.95 | 39.12 |
| SYVKEFHPR | 1 | 1161.59 | 639.60 | 0.00 | 47.19 |
| VAEAINTVEK | 0 | 1072.58 | 0.00 | 1982.17 | 74.17 |
|  |  |  | 6551.3 | 18234.2 |  |
